# Prognostic value of high-sensitivity troponin T levels in patients with ventricular arrhythmias and out-of-hospital cardiac arrest: data from the prospective FINNRESUSCI study

**DOI:** 10.1186/s13054-014-0605-y

**Published:** 2014-11-08

**Authors:** Helge Røsjø, Jukka Vaahersalo, Tor-Arne Hagve, Ville Pettilä, Jouni Kurola, Torbjørn Omland, on behalf of the FINNRESUSCI Laboratory Study Group

**Affiliations:** Division of Medicine, Akershus University Hospital, Sykehusveien 25, Lørenskog, 1478 Norway; K.G. Jebsen Cardiac Research Centre and Center for Heart Failure Research, Institute of Clinical Medicine, University of Oslo, Oslo, Norway; Intensive Care Units, Department of Anaesthesiology, Intensive Care and Pain Medicine, Helsinki University Hospital, Helsinki, Finland; Division of Diagnostics and Technology, Akershus University Hospital, Lørenskog, Norway and Institute of Clinical Medicine, University of Oslo, Oslo, Norway; Centre for Prehospital Emergency Care, Kuopio University Hospital, Kuopio, Finland

## Abstract

**Introduction:**

Myocardial dysfunction is common after out-of-hospital cardiac arrest (OHCA) and high-sensitivity troponin T (hs-TnT) levels may provide incremental prognostic information to established risk indices.

**Methods:**

A total of 155 patients with OHCA and a shockable rhythm (98% ventricular fibrillation; OHCA-VF/VT) had blood samples drawn within six hours of admission. Blood samples were also available after 24 hours, 48 hours, and 96 hours in subsets of patients. The endpoints of the study were hospital mortality and neurological status and mortality after one year.

**Results:**

Admission hs-TnT levels were higher than the 99-percentile of the general population (14 ng/L) in all patients (range 18 to 17837 ng/L). Admission hs-TnT levels were associated with acute coronary artery occlusion, time to return of spontaneous circulation, heart failure, and renal function. Admission hs-TnT levels were higher in one-year non-survivors compared to survivors (median 747 (quartile 1 to 3, 206 to 1061) ng/L versus 345 (184 to 740) ng/L, *P* =0.023) and in patients with a poor versus a favorable neurological outcome (739 (191 to 1061) ng/L versus 334 (195 to 716) ng/L, *P* =0.028). However, hs-TnT measurements did not add prognostic information to established risk variables in multivariate analyses. hs-TnT levels measured during the hospitalization for OHCA-VF/VT correlated closely with admission levels (r ≥0.63) and were inferior to Simplified Acute Physiology Score II (SAPS II) scores for the prediction of events during follow-up. hs-TnT dynamics did not discriminate between survivors and non-survivors or between a poor versus a favorable neurological outcome.

**Conclusion:**

hs-TnT levels are elevated in critically ill patients with OHCA-VF/VT, but do not improve risk prediction.

## Introduction

The long-term morbidity and mortality of successfully resuscitated patients following ventricular arrhythmia-induced out-of-hospital cardiac arrest (OHCA) are still high [1,2]. Standard treatment in patients with OHCA is targeted to support organ function and has included temperature control to alleviate cerebral injury [3], which is known to cause most deaths after OHCA [4-6]. However, as post-cardiac arrest shock affects two thirds of all OHCA patients [5] and contributes to mortality during follow up [4-6], cardiac biomarkers may provide additional prognostic information to established risk factors in OHCA [7]. Early myocardial stunning in post-cardiac arrest shock will also lead to systemic hypotension, which will limit the potential for brain recovery after OHCA [8].

Cardiac-specific troponin I and T are part of the contractile apparatus of cardiomyocytes, but leak into the circulation after cardiomyocyte injury [9]. Thus, symptoms suggestive of myocardial ischemia and dynamic troponin elevations (rise and/or fall) are required for the diagnosis of acute myocardial infarction (AMI) according to the third universal definition of myocardial infarction [10]. Chest pain patients with elevated troponin levels have a worse prognosis than patients with normal troponin levels, but this excessive risk can be reduced by early angiography and percutaneous coronary intervention [11]. Hence, in patients with chest pain troponins are useful for diagnostic and prognostic assessment, as well as for selecting patients who may benefit from invasive therapy. In contrast, whether troponins provide clinically relevant information in patients with ventricular fibrillation or tachycardia (VF/VT) and OHCA has not been established [7]. Previous studies, using sensitive or high-sensitivity (hs) troponin assays suggest that troponins have insufficient accuracy for diagnosing acute coronary artery occlusion in the setting of cardiac arrest [12-14]. However, troponins could still be of interest in OHCA by identifying the patient at highest risk of developing post-cardiac arrest syndrome [8]. To be clinically relevant, studies should be performed in OHCA patients receiving contemporary therapy, including therapeutic hypothermia (TH) [3,8]. Furthermore, as hs troponin assays have been found to be superior to previous generation troponin assays for prognostic assessment across the spectrum of cardiovascular disease [15-18], hs troponin assays should be tested for prognostic utility also in patients with OHCA. In cardiac arrest patients, prognosis differ markedly depending on the initially observed rhythm (shockable versus non-shockable) [19]. Thus, studies assessing the prognostic utility of troponins should preferably encompass a homogeneous patient population; that is, OHCA-VF/VT patients. Accordingly, in this study of OHCA patients with a shockable rhythm we wanted to (1) identify factors that influence hs-TnT levels and (2) assess whether hs-troponin T (hs-TnT) measurements improve prediction of morbidity and mortality, short- and long-term.

## Methods

### Study design

This is a substudy of FINNRESUSCI, an observational prospective multicenter study performed by the Finnish Intensive Care Consortium that comprised 21 of the 22 ICUs in Finland [20]. The study was conducted according to the Declaration of Helsinki and approved by the Ethics Committee of Helsinki University Hospital with written informed consent for blood sampling obtained from a legal representative. Inclusion criteria for the FINNRESUSCI Study were (1) OHCA, (2) successful resuscitation, (3) age >18 y, and (4) post-resuscitation care in a participating ICU.

**Figure 1:**
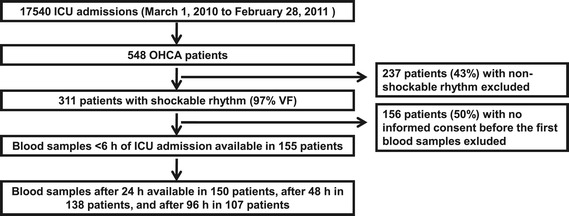
**Flow chart of the study.** OHCA, out-of-hospital cardiac arrest; VF, ventricular fibrillation.

The main study focused on post-resuscitation care for OHCA, and these results have previously been reported [20], including that 86% of unconscious patients with a shockable rhythm were treated with TH (33-34°C). In short, from 1 March 2010 to 28 February 2011 a total of 548 adult patients with OHCA admitted to an ICU were identified. A total of 311 patients had a shockable rhythm (OHCA-VF/VT), and blood samples (after an informed consent) were available within 6 h of ICU admission in 155 (50%) of these patients (Figure 1). No restrictions were implemented regarding techniques of induction or maintenance of TH in the FINNRESUSCI Study. However, the majority of Finnish ICUs use endovascular cooling devices. Patients with a non-shockable rhythm were excluded from this substudy because these patients have a clearly worse prognosis and are subjected to less standardized treatment than patients with a shockable rhythm [19,21].

We collected information on age, gender, weight and height, and previous medical history in an electronic case report form. Body mass index (BMI) was calculated by weight (kg)/(height (m))^2^. We collected information on the cardiac arrest episode, including the time to return of spontaneous circulation (ROSC) according to the Utstein style [22]. We also collected information on treatment during the ICU stay and whether the hospitalization was complicated by pneumonia or sepsis. Coronary angiography was performed at the discretion of the attending physicians at the different centers. We considered coronary artery intervention during the ICU stay as indicative of an acute coronary artery occlusion. The simplified acute physiology score (SAPS) II was calculated 24 h after ICU admission excluding points from temperature and negative effects of sedation on the Glasgow coma scale (GCS). Thus, the best GCS after resuscitation before start of sedation was recorded. We included endpoints of the different phases of the post-cardiac arrest period; that is, hospital mortality, the mortality after 1 year, and neurological status at 1 year after cardiac arrest according to recommendations [8]. Hospital mortality was chosen because SAPS II has been validated for this endpoint. Neurological status was assessed by a structured telephone interview with the patients categorized according to the Pittsburgh cerebral performance categories (CPC) [22]. Patients with CPC scores 1 to 2 were categorized as favorable and CPC scores 3 to 5 as having a poor neurological outcome. Hospital mortality was collected from the medical records and 1-year mortality was obtained from Statistics Finland.

### Biochemical analysis

We measured troponin T in serum samples obtained within 6 h, 24 h, 48 h, and 96 h after cardiac arrest by the Elecsys TNT hs STAT assay (Roche Diagnostics, Penzberg, Germany). The hs-TnT assay has an analytical measurement range of 3 to 10,000 ng/L and the 99th percentile in the healthy population is 14 ng/L. Samples with concentrations above the upper limit were diluted before they were reanalyzed. The analytical characteristics of the hs-TnT assay have previously been reported for our laboratory [23]. Creatinine was measured by routine methods, and we calculated the estimated creatinine clearance by the Cockcroft-Gault formula [24].

### Statistics

We present data as median (quartile (Q) 1 to 3) or absolute numbers and percentages. Continuous variables demonstrated a non-normal distribution, as assessed by the Kolmogorov-Smirnov test, and group differences were explored by the Mann-Whitney *U*-test. Categorical variables were assessed by the Chi-square test or the Fisher exact test. Variables associated with hs-TnT levels on ICU admission were assessed by linear regression analysis and the analysis included age, gender, BMI, estimated creatinine clearance, comorbidities, information on the cardiac arrest, and evidence of acute coronary occlusion. The ability of hs-TnT to predict endpoints during follow up was explored by univariate and multivariate logistic regression analyses. The odds ratios (OR) are presented with 95% CI. Variables significantly associated with the endpoints in univariate analyses were included in the multivariate models (forward selection). hs-TnT levels were transformed by the natural logarithm prior to regression analyses due to a right-skewed distribution. We also assessed prognostic accuracy of hs-TnT levels by receiver operating statistics (ROC) with area under the curve (AUC) presented with 95% CI. We used the Wilcoxon matched-pairs signed-rank test to assess changes in hs-TnT levels (∆) from the initial to later time points. A *P*-value <0.05 was considered statistically significant. Statistical analyses were performed with SPSS for Windows version 19.0 (SPSS, Chicago, IL, USA) and MedCalc for Windows, version 12.1.4.0 (MedCalc Software, Mariakerke, Belgium).

## Results

### Patient characteristics

Characteristics of the patients in this substudy (n =155) were comparable to all patients with OHCA and a shockable rhythm in the main study (n =311) (Table 1). In total, 152 of 155 patients (98%) had ventricular fibrillation as the initial rhythm. The median age was 63 (Q1 to Q3 56 to 72) years, 132 (85%) were male, most patients experienced witnessed cardiac arrest and received bystander cardiopulmonary resuscitation, and 134 (87%) were treated with TH (Table 2).

**Table 1:** **Patient characteristics in the biomarker substudy and the patients with out-of-hospital cardiac arrest** (**OHCA) and a shockable rhythm in the main FINNRESUSCI Study**

	**Biomarker substudy**	**Main study**
	**(n =155)**	**(n =311)**
Age	63 (56 to 72)	63 (56 to 72)
Male sex, n (%)	132 (85%)	253 (81%)
Coronary artery disease, n (%)	50 (32%)	113 (36%)
Diabetes mellitus, n (%)	33 (21%)	59 (19%)
Hypertension, n (%)	70 (45%)	140 (45%)
Heart failure, n (%)	23 (15%)	45 (15%)
SAPS II score	58 (40 to 69)	54 (39 to 66)
**Resuscitation**		
Witnessed cardiac arrest, n (%)	143 (92%)	288 (93%)
Bystander CPR, n (%)	105 (68%)	195 (63%)
Time to ROSC, minutes	20 (14 to 29)	20 (14 to 27)
**Treatment during ICU stay**		
Awake on ICU admission, n (%)	9 (6%)	30 (10%)
Therapeutic hypothermia, n (%)	134 (87%)	241 (78%)
Coronary angiography, n (%)	34 (22%)	72 (23%)
PCI, n (%)	15 (10%)	38 (12%)
**Infection**		
Pneumonia, n (%)	64 (41%)	111 (36%)
Sepsis, n (%)	9 (6%)	14 (5%)
**Outcome**		
Mechanical ventilation, h	50 (40 to 79)	46 (35 to 72)
ICU LOS, days	3.2 (2.2 to 5.0)	2.9 (1.9 to 4.8)
Hospital mortality, n (%)	45 (29%)	96 (31%)
Mortality after 1 year, n (%)	59 (38%)	124 (40%)
CPC 3 to 5 after 1 year, n (%)	65 (42%)	136 (44%)

n, number of patients; hs-TnT, high-sensitivity troponin T; SAPS II, simplified acute physiology score II; CPR, cardiopulmonary resuscitation; ROSC, return of spontaneous circulation; PCI, percutaneous coronary intervention; LOS, length of stay; CPC, cerebral performance categories. Continuous data are presented as median (quartile 1 to 3).

**Table 2:** **Patient characteristics according to hs-TnT levels on inclusion in the study**

	**All patients**	**Quartile (Q) 1 to 2**	**Quartile (Q) 3 to 4**	***P*** **-value**
	**(n =155)**	**(n =78)**	**(n =77)**	**(Q 1 to 2 versus Q 3 to 4)**
hs-TnT, ng/L, range	18-17837	18-415	418-17837	-
Age	63 (56 to 72)	63 (56 to 70)	64 (57 to 72)	0.25
Male sex	132 (85%)	67 (86%)	65 (84%)	0.80
Body mass index	26 (24 to 29)	27 (25 to 29)	26 (24 to 28)	0.054
Creatinine clearance, mL/minute	94 (72 to 126)	100 (77 to 129)	88 (67 to 123)	0.13
Coronary artery disease, n (%)	50 (32%)	26 (33%)	24 (31%)	0.77
Diabetes mellitus, n (%)	33 (21%)	22 (28%)	11 (14%)	0.034
Hypertension, n (%)	70 (45%)	35 (45%)	35 (46%)	0.94
Heart failure, n (%)	23 (15%)	16 (21%)	7 (9%)	0.045
SAPS II score	58 (40 to 69)	54 (37 to 66)	62 (44 to 69)	0.034
**Resuscitation**				
Witnessed cardiac arrest, n (%)	143 (92%)	73 (94%)	70 (91%)	0.53
Bystander CPR, n (%)	105 (68%)	55 (71%)	50 (65%)	0.46
Time to ROSC, minutes	20 (14 to 29)	17 (11 to 21)	26 (18 to 32)	<0.001
Treatment during ICU stay				
Therapeutic hypothermia	134 (87%)	67 (86%)	67 (87%)	0.84
Coronary angiography	34 (22%)	14 (18%)	20 (26%)	0.23
PCI	15 (10%)	2 (3%)	13 (17%)	0.003
**Infection**				
Pneumonia	64 (41%)	34 (44%)	30 (39%)	0.56
Sepsis	9 (6%)	5 (6%)	4 (5%)	1.00
**Outcome**				
Mechanical ventilation, h	50 (40 to 79)	49 (40 to 73)	57 (43 to 89)	0.24
ICU LOS, days	3.2 (2.2 to 5.0)	3.1 (2.1 to 5.2)	3.6 (2.3 to 4.9)	0.51
Hospital mortality	45 (29%)	18 (23%)	27 (35%)	0.10
Mortality after 1 year	59 (38%)	22 (28%)	37 (48%)	0.011
CPC 3 to 5 after 1 year	65 (42%)	24 (31%)	41 (53%)	0.005

n, number of patients; hs-TnT, high-sensitivity troponin T; SAPS II, simplified acute physiology score II; SOFA, sequential organ failure assessment score CPR, cardiopulmonary resuscitation; ROSC, return of spontaneous circulation; PCI, percutaneous coronary intervention; LOS, length of stay; CPC, cerebral performance categories. Continuous data are presented as median (quartile 1 to 3).

### Admission hs-TnT levels

The range of hs-TnT levels within 6 h of admission for OHCA-VF/VT was 18 to 17,837 ng/L with median level 415 ng/L (Q1 to Q3 199 to 916 ng/L). The prevalence of diabetes mellitus and heart failure was higher among patients with hs-TnT levels below the median, while time to ROSC was longer in patients with supramedian hs-TnT levels (Table 2). The median hs-TnT level in patients with acute coronary artery occlusions (n =15) was 1,497 ng/L (Q1 to Q3 753 to 8,875 ng/L) compared to median 387 ng/L (182 to 815 ng/L) for the other patients (*P* <0.001). Admission hs-TnT levels were not associated with the probability of receiving TH (Table 2).

Admission hs-TnT levels correlated positively with time to ROSC (*r* =0.47, *P* <0.001), SAPS II score (*r* =0.16, *P* =0.045), and duration of mechanical ventilation (*r* =0.17, *P* =0.038), and were inversely correlated to BMI and estimated creatinine clearance (both *r* = -0.17, *P* =0.032). Evidence of acute coronary artery occlusion (β =0.39, *P* <0.001), time to ROSC (β =0.35, *P* <0.001), history of heart failure (β = -0.22, *P* =0.001), and estimated creatinine clearance (β = -0.17, *P* =0.013) were associated with admission hs-TnT levels in multivariate linear regression analysis (*r*^2^ = 0.37). We found close correlations between admission hs-TnT levels and hs-TnT levels after 24 h (*r* =0.78); after 48 h (*r* =0.71); and after 96 h (*r* =0.63) (*P* <0.001 for all).

### hs-TnT levels and hospital and 1-year mortality/poor neurological outcome (CPC 3 to 5)

Of the 155 patients, 45 patients (29%) died during the hospitalization. One year after cardiac arrest 59 patients (38%) had died and 65 patients (42%) had a poor neurological outcome (CPC 3 to 5). Admission hs-TnT levels were not statistically different between hospital non-survivors and hospital survivors: 792 ng/L (191 to 1224 ng/L) versus 387 ng/L (195 to 756 ng/L), respectively, *P* =0.08. In contrast, admission hs-TnT levels were higher in 1-year non-survivors versus survivors (747 ng/L (206 to 1061 ng/L) versus 345 ng/L (184 to 740 ng/L) *P* =0.023) and in patients with a poor neurological outcome compared to a favorable neurological outcome (739 ng/L (191 to 1061 ng/L) versus 334 ng/L (195 to 716 ng/L), *P* =0.028). As assessed by ROC curve analysis, admission hs-TnT levels separated between patients that were dead or categorized as having an unfavorable neurological outcome after 1 year, but did not discriminate regarding hospital mortality (Table 3). Similar results were also found for hs-TnT measurements after 24 h, but the accuracy of hs-TnT levels after 24 h were inferior to SAPS II score for all endpoints (Table 4).

**Table 3:** **Prognostic value of high-sensitivity troponin T (hs-TnT) measured ≤24 h after ICU admission as assessed by receiver operating characteristic curve analysis**

	**Hospital mortality**			**Mortality after 1 year**			**CPC 3 to 5 after 1 year**		
	**AUC**	**95% CI**	***P*** **-value**	**AUC**	**95% CI**	***P*** **-value**	**AUC**	**95% CI**	***P*** **-value**
**Admission (n =155)**	0.59	0.51, 0.68	0.10	0.61	0.52, 0.68	0.03	0.60	0.52, 0.68	0.03
**24 h (n =150)**	0.60	0.51, 0.68	0.05	0.62	0.54, 0.70	0.009	0.62	0.54, 0.70	0.01
**∆ hs-TnT (n =150)**	0.50	0.42, 0.58	0.99	0.51	0.43, 0.59	0.85	0.51	0.42, 0.59	0.92

CPC, cerebral performance categories; AUC, area under the curve; ∆ (delta), change in hs-TnT levels from admission to 24 h.

**Table 4:** **Comparison of high-sensitivity troponin T (hs-TnT) measured ≥24 h after ICU admission and SAPS II score as assessed by receiver operating characteristics curve analysis**

	**Hospital mortality**			**Mortality after 1 year**			**CPC 3 to 5 after 1 year**		
	**AUC**	**95% CI**	***P*** **-value**	**AUC**	**95% CI**	***P*** **-value**	**AUC**	**95% CI**	***P*** **-value**
**After 24 h(n =150)**									
SAPS II score	0.76	0.69, 0.83	Reference	0.76	0.69, 0.83	Reference	0.78	0.70, 0.84	Reference
hs-TnT	0.60	0.51, 0.68	0.005	0.62	0.54, 0.70	0.02	0.62	0.54, 0.70	0.004
∆ hs-TnT	0.50	0.42, 0.58	<0.001	0.51	0.43, 0.59	<0.001	0.51	0.42, 0.59	<0.001
**hs-TnT, 48 h (n =138)**									
SAPS II score	0.76	0.68, 0.83	Reference	0.77	0.69, 0.83	Reference	0.78	0.70, 0.85	Reference
hs-TnT	0.63	0.54, 0.71	0.03	0.65	0.56, 0.73	0.04	0.66	0.57, 0.74	0.03
∆ hs-TnT	0.54	0.45, 0.62	0.006	0.53	0.44, 0.61	<0.001	0.51	0.42, 0.59	<0.001
**hs-TnT, 96 h (n =107)**									
SAPS II score	n.a.			0.75	0.68, 0.82	-	0.79	0.70, 0.86	Reference
hs-TnT	n.a.			0.62	0.54, 0.70	0.02	0.60	0.50, 0.69	0.005
∆ hs-TnT	n.a.			0.51	0.43, 0.59	<0.001	0.53	0.43, 0.63	0.001

*P*-values are for simplified acute physiology score (SAPS) II versus high-sensitivity troponin T **(**hs-TnT) levels. CPC, cerebral performance categories; We did not calculate results for hospital mortality for measurements after 96 h due to the low number of events in this group (n =17) (total hospital mortality in the FINNRESUSCI laboratory substudy was 45 patients). AUC, area under the curve; ∆ (delta), change in hs-TnT levels from admission to later timepoints; n.a., not applicable.

**Figure 2:**
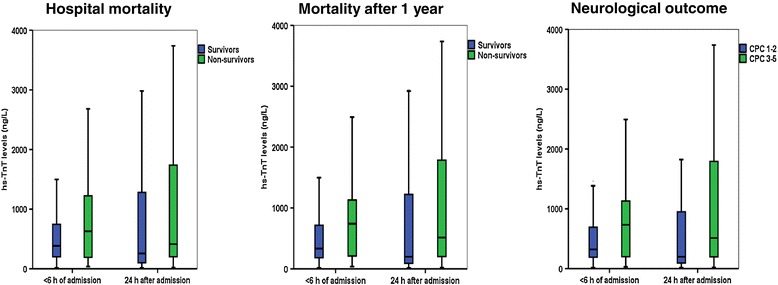
**hs-TnT levels on admission and after 24 h in patients with OHCA-VF/VT divided according to mortality and neurological outcome.** The horizontal line within the box represents the median concentration, the boundaries of the box quartiles 1-3, and the whiskers range (maximum value restricted to 1.5 x interquartile range from the median).

**Figure 3:**
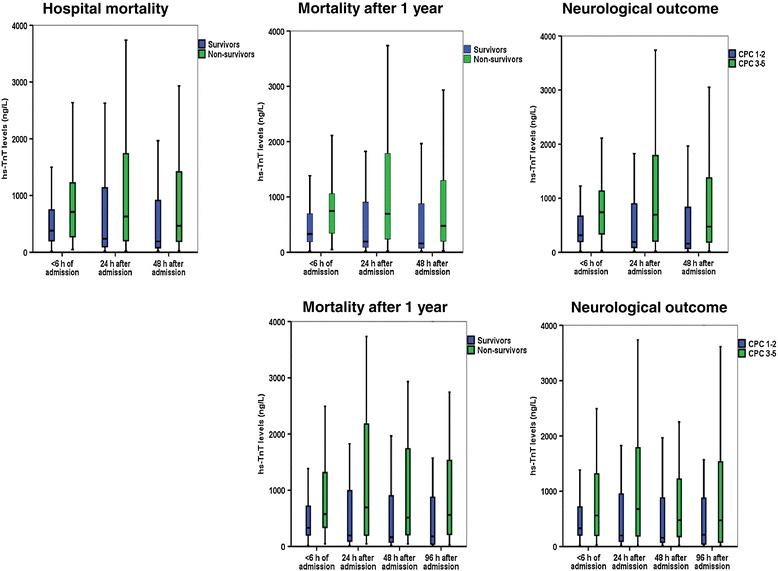
**High-sensitivity troponin T (hs-TnT) levels in the patients with blood samples available also after 48 h (upper panel) and 96 h (lower panel).** The horizontal line within the box represents the median concentration, the boundaries of the box quartiles 1 to 3, and the whiskers range (maximum value restricted to 1.5 × interquartile range from the median).

Most patients demonstrated a reduction in hs-TnT levels from admission to 24 h (∆ hs-TnT levels), but no significant differences were found for ∆ hs-TnT levels relating to hospital mortality (median 10 versus 51 ng/L reduction for non-survivors versus survivors); 1-year mortality (68 versus 46 ng/L reduction); nor neurological outcome (median 46 versus 49 ng/L reduction for poor versus favorable outcome) (Figure 2). In most patients, hs-TnT levels further decreased 48 h and 96 h after ICU admission with no differences in hs-TnT dynamics according to mortality or morbidity during follow up (Table 4 and Figure 3). By logistic regression analyses, admission hs-TnT levels (logarithmically transformed) were neither associated with hospital nor 1-year mortality, nor with 1-year neurological outcome according to CPC score (Table 5). In contrast, we found several established risk variables in OHCA-VF/VT associated with mortality and morbidity in our cohort (Table 5).

**Table 5:** **Associations between variables available <24 h after admission for OHCA-VF/VT and hospital mortality and 1 year outcomes**

	**Hospital mortality**			**Mortality after 1 year**			**CPC 3 to 5 after 1 year**		
	**Odds ratio**	**95% CI**	***P*** **-value**	**Odds ratio**	**95% CI**	***P*** **-value**	**Odds ratio**	**95% CI**	***P*** **-value**
**Univariate analysis**									
Age	1.05	1.02, 1.09	0.003	1.05	1.02, 1.08	0.002	1.05	1.02, 1.08	0.001
Male	1.19	0.44, 3.24	0.74	1.90	0.70, 5.13	0.21	1.79	0.69, 4.64	0.23
Body mass index	1.006	0.93, 1.09	0.89	1.004	0.94, 1.08	0.91	0.99	0.93, 1.07	0.87
Creatinine clearance	0.99	0.98, 0.99	0.011	0.99	0.98, 1.00	0.052	0.99	0.98, 1.00	0.062
History of coronary artery disease	2.80	1.36, 5.78	0.005	2.68	1.34, 5.36	0.005	2.65	1.33, 5.28	0.006
History of diabetes mellitus	2.56	1.15, 5.69	0.021	1.48	0.68, 3.22	0.33	1.20	0.55, 2.60	0.64
History of hypertension	1.40	0.70, 2.81	0.34	1.16	0.61, 2.23	0.65	1.07	0.56, 2.03	0.83
History of heart failure	3.27	1.32, 8.12	0.010	3.01	1.21, 7.48	0.018	3.08	1.22, 7.77	0.018
Witnessed cardiac arrest	0.38	0.11, 1.23	0.11	0.28	0.08, 0.97	0.044	0.22	0.06, 0.83	0.025
Bystander CPR	0.93	0.45, 1.95	0.86	0.89	0.44, 1.77	0.73	0.78	0.40, 1.54	0.48
Time to ROSC	1.07	1.03, 1.10	<0.001	1.09	1.05, 1.13	<0.001	1.09	1.05, 1.13	<0.001
Therapeutic hypothermia	0.79	0.30, 2.11	0.64	1.27	0.48, 3.35	0.63	1.20	0.47, 3.10	0.70
Coronary angiography	0.26	0.09, 0.79	0.017	0.28	0.11, 0.71	0.008	0.28	0.11, 0.70	0.006
PCI	0.16	0.02, 1.22	0.077	0.10	0.01, 0.79	0.029	0.09	0.01, 0.66	0.019
hs-TnT <6 h	1.25	0.95, 1.64	0.12	1.26	0.97, 1.63	0.09	1.26	0.97, 1.63	0.08
**Multivariate analysis**									
Age	1.07	1.03, 1.11	0.001	1.05	1.02, 1.09	0.004	1.06	1.02, 1.09	0.003
Time to ROSC	1.07	1.03, 1.11	<0.001	1.11	1.06, 1.16	<0.001	1.11	1.07, 1.16	<0.001
Diabetes mellitus	2.87	1.19, 6.95	0.019	n.s.			n.s.		
PCI	n.s.			0.09	0.01, 0.79	0.030	0.07	0.007, 0.61	0.017

OHCA, out-of-hospital cardiac arrest; VF, ventricular fibrillation; VT, ventricular tachycardia; CPR, cardiopulmonary resuscitation; hs-TnT, high-sensitivity troponin T ROSC, return of spontaneous circulation; PCI, percutaneous coronary intervention; n.s., non-significant.

## Discussion

The main finding of this study was that hs-TnT levels were above the 99th percentile in all critically ill patients successfully resuscitated from OHCA after VF or VT. However, hs-TnT measurements on admission or later during the ICU stay did not provide incremental prognostic information about hospital mortality or 1-year neurological outcome. hs-TnT dynamics during the ICU stay also failed to provide prognostic information, and the lack of prognostic information obtained from hs-TnT measurement was evident for both hospital and 1-year mortality.

Several factors may explain the limited prognostic information by measuring hs-TnT levels in patients with OHCA-VF/VT. First, myocardial cell necrosis is not the main determinant of outcome after cardiac arrest [4-6]. Hence, in patients with OHCA-VF/VT other factors such as brain injury and the systemic ischemia/reperfusion syndrome will be of greater importance for short- and long-term outcome [7]. Deaths during follow up may also relate to the initial brain injury, for example, patients with poor cognitive function after OHCA-VF/VT will be susceptible to dying from pneumonia or other infections during follow up. Thus, unlike the situation in chest-pain patients, pathology not reflected by hs-TnT levels is likely to determine survival after cardiac arrest.

Second, the influence of several factors besides acute coronary artery occlusion to hs-TnT levels may attenuate the prognostic value of hs-TnT in OHCA-VF/VT patients. The duration of cardiopulmonary resuscitation and number of defibrillation shocks have previously been reported to increase troponin levels [25-27], and we also found the time to ROSC to be closely associated with admission hs-TnT levels. Impaired renal function was also associated with increased hs-TnT levels in OHCA-VF/VT patients and this association is well-known from previous studies [16-18,23]. In contrast, it is surprising that a previous diagnosis of heart failure was associated with lower hs-TnT levels in our study as patients with heart failure in general will have high troponin levels [28]. A possible explanation for this result could be less concomitant acute coronary artery occlusion in heart failure patients with OHCA-VF/VT, thus, heart failure patients with OHCA-VF/VT will have elevated hs-TnT levels compared to the general population, but still lower levels compared to other patients with OHCA-VF/VT. The very high hs-TnT levels observed in this study support this model. Previous studies using older troponin assays have also found troponin levels to exceed the upper reference limit in the majority of OHCA patients [29-31].

We now support and extend these observations by reporting hs-TnT levels above the 99th percentile of the healthy population in all patients with OHCA-VF/VT. The high troponin levels in OHCA-VF/VT patients are likely a result of prolonged cardiopulmonary resuscitation, multiple defibrillation shocks, and a proportion of the patients having acute coronary artery occlusion as the cause of OHCA-VF/VT. Of note, the results for hs-TnT in OHCA-VF/VT are analogous to the situation for hs-TnT measurements in other cohorts of critically ill patients, including in severe sepsis where hs-TnT levels are elevated in the majority of patients, but fail to improve risk assessment beyond established risk indices [32].

Our study has several strengths. First, an obvious strength is that the OHCA-VF/VT patients in this biomarker substudy were comparable to the OHCA-VF/VT patients in the large observational FINNRESUSCI study and should therefore be representative of the general OHCA-VF/VT cohort. In addition, a high proportion of patients (86%) were treated with TH according to the current guidelines. These two issues increase the external validity of our findings. We also measured troponin T levels at several time points by a high-sensitivity assay, which allowed us to assess the relevance of troponin dynamics. However, any inferences based on the findings of this study are subject to some obvious limitations. First, we were not able to study consecutive patients due to the requirement of written informed consent for blood sampling. Second, the majority of our patients were not examined by angiography, although the proportion in our study were comparable to the proportion examined by angiography in the main FINNRESUSCI Study [20] and in other studies [29,31]. Our study was not designed to assess the potential of hs-TnT to diagnose acute coronary occlusion and hs-TnT levels could still be of value for selecting patients who may benefit from coronary revascularization in OHCA-VF/VT. Furthermore, other cardiac biomarkers like the B-type natriuretic peptides reflect additional pathophysiology and may provide stronger prognostic information in OHCA-VF/VT than hs-TnT levels. Finally, the study patients were treated according to the current guidelines proposing TH (33 to 34°C), and the results may be different in future populations if milder hypothermia (36°C) will be recommended [33].

## Conclusion

We found hs-TnT levels to exceed the 99th percentile in successfully resuscitated OHCA-VF/VT patients admitted to the ICU. However, our data do not support the use of hs-TnT measurements for risk stratification in critically ill OHCA-VF/VT patients.

## Key messages

- hs-TnT levels were elevated in all patients with OHCA-VF/VT
- Admission hs-TnT levels were higher in 1-year non-survivors and patients with an unfavorable neurological outcome after 1 year
- hs-TnT measurements did not add to current risk variables in OHCA-VF/VT.

## Abbreviations

AMI: acute myocardial infarction
AUC: area under the curve
BMI: body mass index
CPC: cerebral performance categories
GCS: Glasgow coma scale
hs-TnT: high-sensitivity troponin T levels
OHCA: out-of-hospital cardiac arrest
OR: odds ratio
PCP: Pittsburgh cerebral performance categories
Q: quartile
ROC: receiver operating characteristic statistics
ROSC: return of spontaneous circulation
SAPS II: simplified acute physiology score II
TH: therapeutic hypothermia
VF: ventricular fibrillation
VT: ventricular tachycardia
